# Synchronization of inspiratory burst onset along the ventral respiratory column in the neonate mouse is mediated by electrotonic coupling

**DOI:** 10.1186/s12915-023-01575-5

**Published:** 2023-04-15

**Authors:** Boris Gourévitch, Teresa Pitts, Kimberly Iceman, Mitchell Reed, Jun Cai, Tianci Chu, Wenxin Zeng, Consuelo Morgado-Valle, Nicholas Mellen

**Affiliations:** 1 Unité de Génétique Et Physiologie de L’Audition, INSERM, Institut Pasteur, Sorbonne Université, 75015 Paris, France; 2 Department of Neurological Surgery, University of Louisville, Louisville, KY USA; 3 Department of Pediatrics, University of Louisville, Louisville, KY USA; 4 Instituto de Investigaciones Cerebrales, Universidad Veracruzana, Xalapa, Veracruz, México; 5 Department of Neurology, University of Louisville, Louisville, KY USA

## Abstract

Breathing is a singularly robust behavior, yet this motor pattern is continuously modulated at slow and fast timescales to maintain blood-gas homeostasis, while intercalating orofacial behaviors. This functional multiplexing goes beyond the rhythmogenic function that is typically ascribed to medullary respiration-modulated networks and may explain lack of progress in identifying the mechanism and constituents of the respiratory rhythm generator. By recording optically along the ventral respiratory column in medulla, we found convergent evidence that rhythmogenic function is distributed over a dispersed and heterogeneous network that is synchronized by electrotonic coupling across a neuronal syncytium. First, high-speed recordings revealed that inspiratory onset occurred synchronously along the column and did not emanate from a rhythmogenic core. Second, following synaptic isolation, synchronized stationary rhythmic activity was detected along the column. This activity was attenuated following gap junction blockade and was silenced by tetrodotoxin. The layering of syncytial and synaptic coupling complicates identification of rhythmogenic mechanism, while enabling functional multiplexing.

**Supplementary Information:**

The online version contains supplementary material available at 10.1186/s12915-023-01575-5.

## Background

Networks essential for breathing in mammals are distributed along the ventral respiratory column (VRC) running rostrocaudally in ventrolateral medulla [1, 2]. In vitro preparations isolated from neonate rodents spontaneously generate respiratory rhythm [3]. The minimal network sufficient for rhythmogenesis was isolated in the transverse slice preparation containing the pre-Bötzinger complex (preBötC) [4], but in the ensuing decades, the taxonomy, connectivity, and mechanism for respiratory rhythm generation have remained elusive [5]. The aim of this study was to elucidate respiratory rhythmogenesis by contextualizing preBötC activity within the larger ventral respiratory column using the sagittally sectioned rodent hindbrain preparation (SSRH) [6–8], which exposes the VRC along its major axis. Respiratory activity was recorded optically in a field of view encompassing relay networks caudal to preBötC, as well as the putatively rhythmogenic parafacial respiratory group/retrotrapezoid nucleus (pFRG/RTN) at the ventrolateral margin of the facial nucleus (VIIn) [9], and the postinspiratory complex (PiCo) dorsomedial to VIIn [10], both 400 μm rostral to preBötC.

High-speed optical recordings of intact network activity over long recording epochs and slow, high-sensitivity optical recordings following synaptic blockade provided unexpected but mechanistically linked results. At the network level, neurons active earliest in inspiration were dispersed along the VRC. Tightly synchronized activation at inspiratory onset along the VRC was confirmed via paired macropatch recordings. This finding is incompatible with the hypothesis that respiratory rhythm is generated by an anatomically compact rhythmogenic network. Tight synchronization over such a dispersed network is also difficult to account for via synaptic signaling in an only partially myelinated network. Optical recordings following synaptic blockade provided evidence for a mechanism for tight synchronization over a dispersed network: low-amplitude tightly synchronized, stationary, and anatomically diffuse oscillatory activity was detected. The dynamics and frequency of this activity correspond well with burstlets recorded in the transverse slice [11], and its anatomical dispersion is consistent with gap junction coupling across a syncytium. Because the syncytial oscillations revealed by synaptic blockade were attenuated or blocked by gap-junction blockers at a concentration that silences respiratory activity in the synaptically intact network, gap junctions are the likely mechanism for coupling across the syncytium. Several lines of evidence suggest neurons are necessary constituents of the syncytium: patch-clamp recordings revealed oscillatory fluctuations synchronized with oscillations obtained during concurrent optical recordings; syncytial oscillations were detected in optical recordings of exclusively neuronal source; and syncytial oscillations were eliminated by tetrodotoxin. Because syncytial oscillations were also observed in recordings from glia only, it is likely that glia also participate in syncytial rhythm propagation. Although the mechanism giving rise to syncytial oscillations, and their role in respiratory rhythmogenesis is not addressed this study, these findings support the conjecture that inspiratory burst onsets are synchronized over an anatomically dispersed network along the VRC and that synchronization along this network is mediated by a neuronal gap junction-coupled syncytium.

## Results

**Fig. 1:**
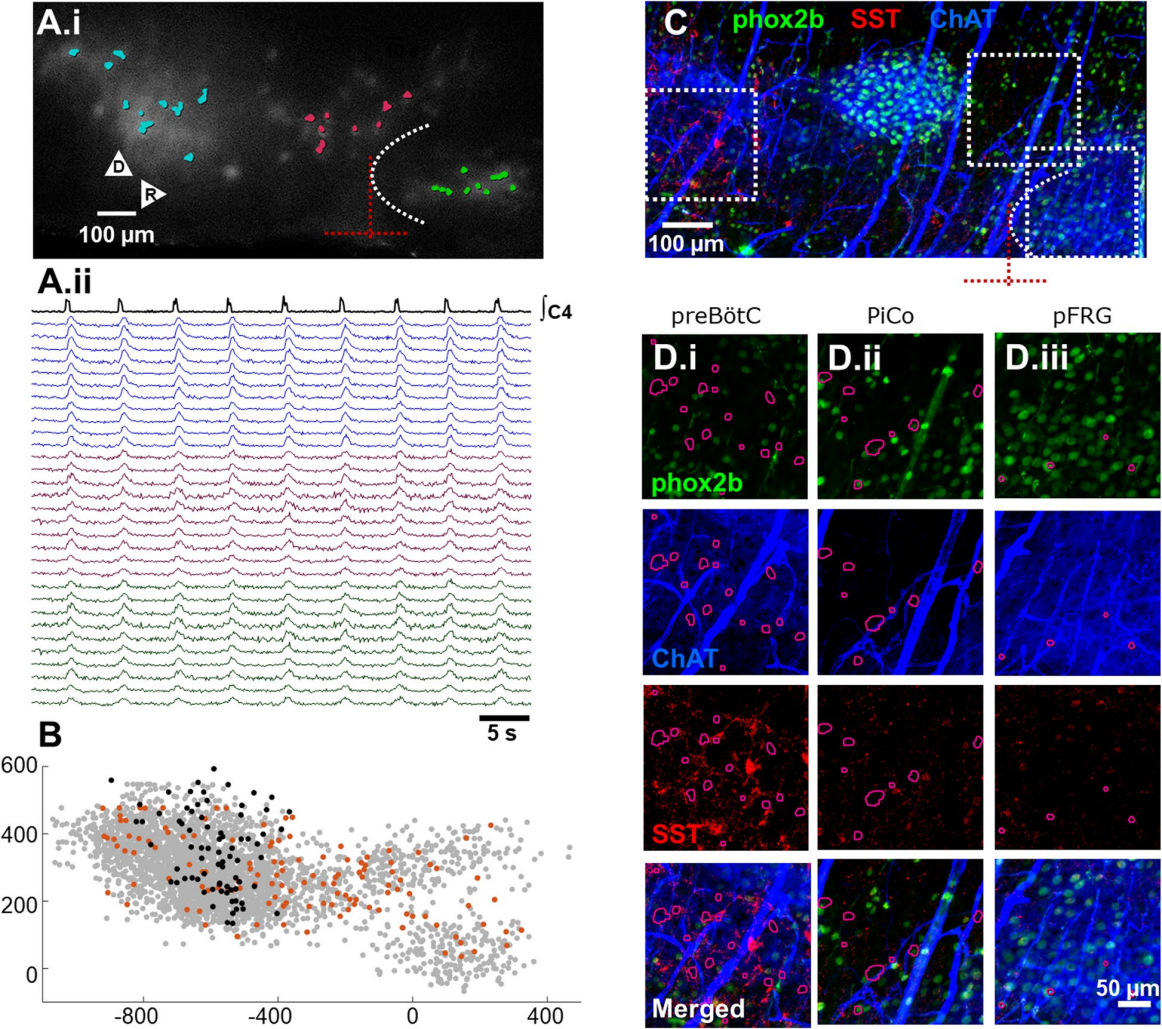
Features of the preparation. **A.i** The average of network activity during individual inspiratory burst reveals synchronized activity along the VRC; salience of Ca^2+^ signals are enhanced by subtracting each image from the image acquire 1.7 s earlier. Intersecting dashed red lines indicate the origin used to align datasets, defined as the caudal pole of the VIIn (dashed white arc) and the minimum of the ventral surface concavity caudal to VIIn. ROIs overlapping with preBötC (blue), PiCo (red), and pFRG/RTN (green) are superimposed **A.ii** Traces associated ROIs in preBötC (blue traces), in PiCo (red traces), and pFRG/RTN (green traces) reveals stereotypy of motor output along the VRC. **B** ROI centers of mass from one germline-GCaMP6F dataset (orange points) and an inducible-Cre dbx1-GCaMP6F exposed to tamoxifen at E9.5 to selectively express GCaMP6F in preBötC neurons (black points) are overlayed on collated data from 180 s (*N* = 11, 1285 ROIs) and 600 s (*N* = 11, 1534 ROIs) high-speed (100 Hz) optical recordings, shown in gray. **C** Merged maximum intensity images obtained from a 450 μm slice sectioned from the preparation shown in **A**, processed for phox2b (green), SST (red), and ChAT (blue). After rescaling, immunoprocessed and fresh tissue were aligned using optical signals and vasculature (video in Additional file 1). **D** ROIs superimposed on images from immunoprocessed tissue reveal preBötC ROIs are in a region with sparse phox2b and ChAT expression but high SST expression (**D.i**). By contrast, PiCo ROIs are located in a region with few phox2b^+^ ChAT^+^ or SST^+^ neurons (**D.ii**). ROIs in pFRG/RTN overlapped with phox2b^+^ and ChAT^+^ networks but overlap with SST^+^ neurons was low (**D.iii**)

Optical recordings were made from neonate mice in which germline expression [12] of the genetically encoded Ca^2+^ indicator GCaMP6F was obtained by crossing male B6.Cg-Tg(Camk2a-cre)T29-1Stl/J or female B6.Cg-Edil3Tg(Sox2-cre)1Amc/J mice with B6J.Cg-Gt(ROSA)26Sortm95.1(CAG-GCaMP6f)Hze/MwarJ mice. In the SSRH preparation, stable respiratory activity could be simultaneously recorded from the preBötC, PiCo and pFRG/RTN (Fig. 1A.i; Video in Additional file 1). Optical recording along the VRC revealed synchronized activity during inspiration (Fig. 1A.ii). Using the caudal pole of VIIn and the ventral surface minimum as an origin (dashed red lines Fig. 1A.i, C), data were pooled across experiments, revealing clustering in each respiratory region (Fig. 1B). By sectioning off the sagittal face for immunoprocessing and bringing fresh and fixed tissue images into register, we were able to confirm overlaps between somatostatin (SST) expression and preBötC networks (Fig. 1D.i) and phox2b expression and pFRG/RTN networks (Fig. 1D.iii; Video in Additional file 1), consistent with published reports [13, 14]. Choline acetyltransferase (ChAT) expression showed only weak overlap with PiCo networks (Fig. 1D.ii), likely because ChAT^+^ PiCo constituents are more medial [10]. Taken together these findings confirm that preBötC, PiCo and pFRG/RTN form spatially segregated pools of respiration-modulated neurons that can be recorded in parallel, enabling a mesoscopic description of network dynamics along the VRC with single-neuron resolution. Further, IHC enhances replicability, because optically recorded dynamics are sensitive to level of section, and level of section can be estimated precisely based on immunohistochemical expression patterns.

### Inspiratory bursts do not propagate from a spatially compact region

**Fig. 2:**
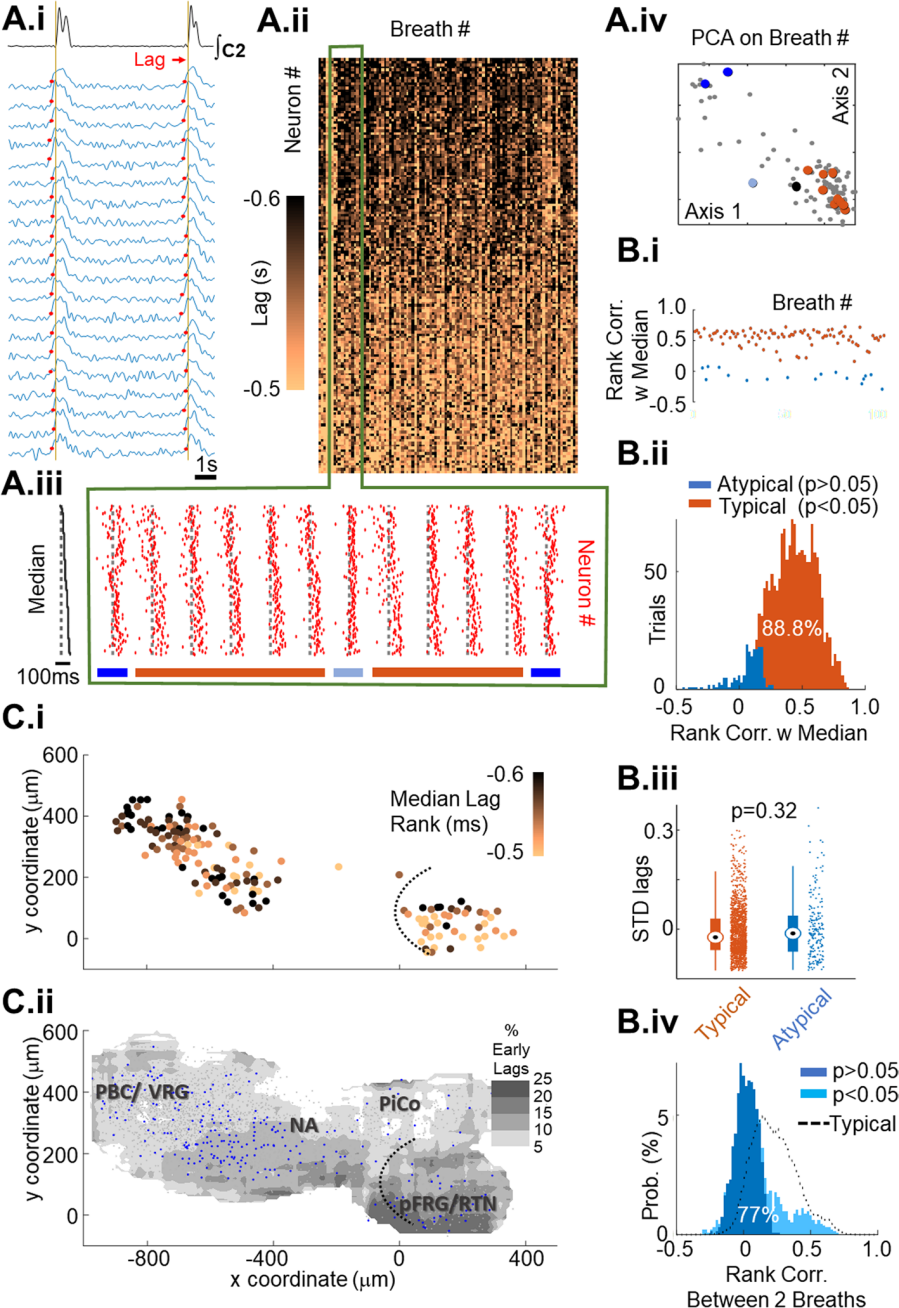
Neuronal onsets are tightly synchronized along the VRC. **A.i** Lag was estimated as the interval between steep luminance rise in individual somatic Ca^2+^ transients and rise in the rectified integrated motor output. **A.ii** Heat map of neuronal lags ordered according to median ranking of onset times over 102 respiratory cycles. A clear gradient from black to beige highlights the fact that most cycles approximated the median ordering. Onset ordering was qualitatively different in a subset of inspiratory bursts indicated by contrasting vertical stripes. **A.iii** Analysis of ranked onset times contained in the green rectangle in **A.ii**. Raster plot of onset times ordered according to the median activation sequence; cycles significantly correlated with median ranking of onsets (“Typical,” orange bars) were interspersed with atypical onset sequences (light and dark blue bars). **A.iv** Principal component analysis reveals that typical lag rankings clustered (lower right), but a smaller cluster associated with alternate lag rankings indicates that alternate rankings also displayed stereotypy. Colored dots indicate typical and atypical breaths shown in A.iii. Black dot indicates median lag rank. **B.i** Rank correlations of individual inspiratory burst onset ordering with median rank ordering. Cycles whose ordering is significantly different are shown as blue points. **B.ii** Pooled data from 22 experiments reveals that most (88.8%, orange histogram) inspiratory onset sequences were not significantly different from their respective median ranking of lags. **B.iii** Standard deviations of typical and atypical inspiratory bursts are not significantly different, suggesting that atypical inspiratory bursts did not result from failed network synchronization. **B.iv** Most (77%) of the 11.2% of cycles categorized as alternate are uncorrelated with one another. **C.i** Anatomical location of earliest neurons for the experiment shown in **A** (lags color-coded as in **A.ii**) reveals that they span from pFRG/RTN to caudal preBötC. **C.ii** Spatial distribution of the 10% earliest ROIs (blue points) reveals that while a slightly higher proportion of earliest ROIs were located in the vicinity of pFRG/RTN, earliest ROIs were distributed along the entire ventral respiratory column. PBC/VRG, preBötC/ventral respiratory group; NA, nucleus ambiguus; PiCo, post-inspiratory complex; pFRG/RTN, parafacial respiratory group/retrotrapezoid nucleus

The first set of experiments sought to exploit the inclusive access to respiratory networks afforded by germline expression of GCaMP6F to identify the locus of inspiratory burst onset. This was based on the premise that if a spatially compact central pattern generator for breathing was located along the VRC, then inspiratory bursts would initiate there. To this end, we carried out optical recordings that were analyzed using semi-automated methods [15] to extract regions of interest (ROIs). Because images were obtained using widefield microscopy, a given ROI may be associated with somatic Ca^2+^ transients from multiple neurons or glia at different depths in the tissue. To convey this ambiguity, we refer to ROIs to denote local neuronal/glial activity. Recordings were made at a sampling rate fast enough (100 Hz) to capture the dispersion of firing onsets during inspiratory bursts (Video in Additional file 2; Fig. 2A.i), over recording epochs of 180 s (*N* = 11, 1285 ROIs) and 600 s (*N* = 11, 1534 ROIs; pooled data are shown in Fig. 1B).

ROI burst onset times relative to motor output (lag) were estimated from traces of time-varying luminance values (Fig. 2A.i) and displayed in animated form for the same dataset shown in Additional File 2 (animation shown in Additional file 3). For each burst, ROIs were sorted into onset sequences from earliest to last, in relation to motor output. From burst to burst, onset sequences varied; to describe onset times over all the bursts in a recording bout, the median lag across all bursts was estimated for each ROI. ROIs were then sorted on their median lag rank from earliest (black) to latest (pale orange) to generate a heat-map of onset times (Fig. 2A.ii). Data from one experiment illustrate common features seen across experiments. Typical onset sequences were defined as sequences that significantly correlated with the median onset sequence (orange bars in Fig. 2A.iii bottom; orange points in Fig. 2B.i). Typical onset sequences were observed in 88.8% of all inspiratory bursts (Fig. 2B.ii). Atypical onset sequences were defined as sequences differing significantly from the median sequence; these are shown as dark stripes in the heat-map of onset times (A.ii) and are indicated by blue bars (Fig. 2A.iii bottom) or as blue points (Fig. 2A.iv, B.i). Two observations undermine the conjecture that atypical onset sequences are ectopic bursts arising out of network failure: the standard deviation of lags in atypical cycles was not significantly different from lags in typical cycles (Fig. 2B.iii); in a subset of experiments, atypical cycle onset sequences were correlated with one another to form distinct modes (23%) (Fig. 2B.iv). Atypical onset sequences, though few in number, occurred in all 22 experiments, suggesting that inspiratory bursts do not arise out of anatomically specified network.

### Macropatch recordings corroborate synchronous activation along the VRC

**Fig. 3:**
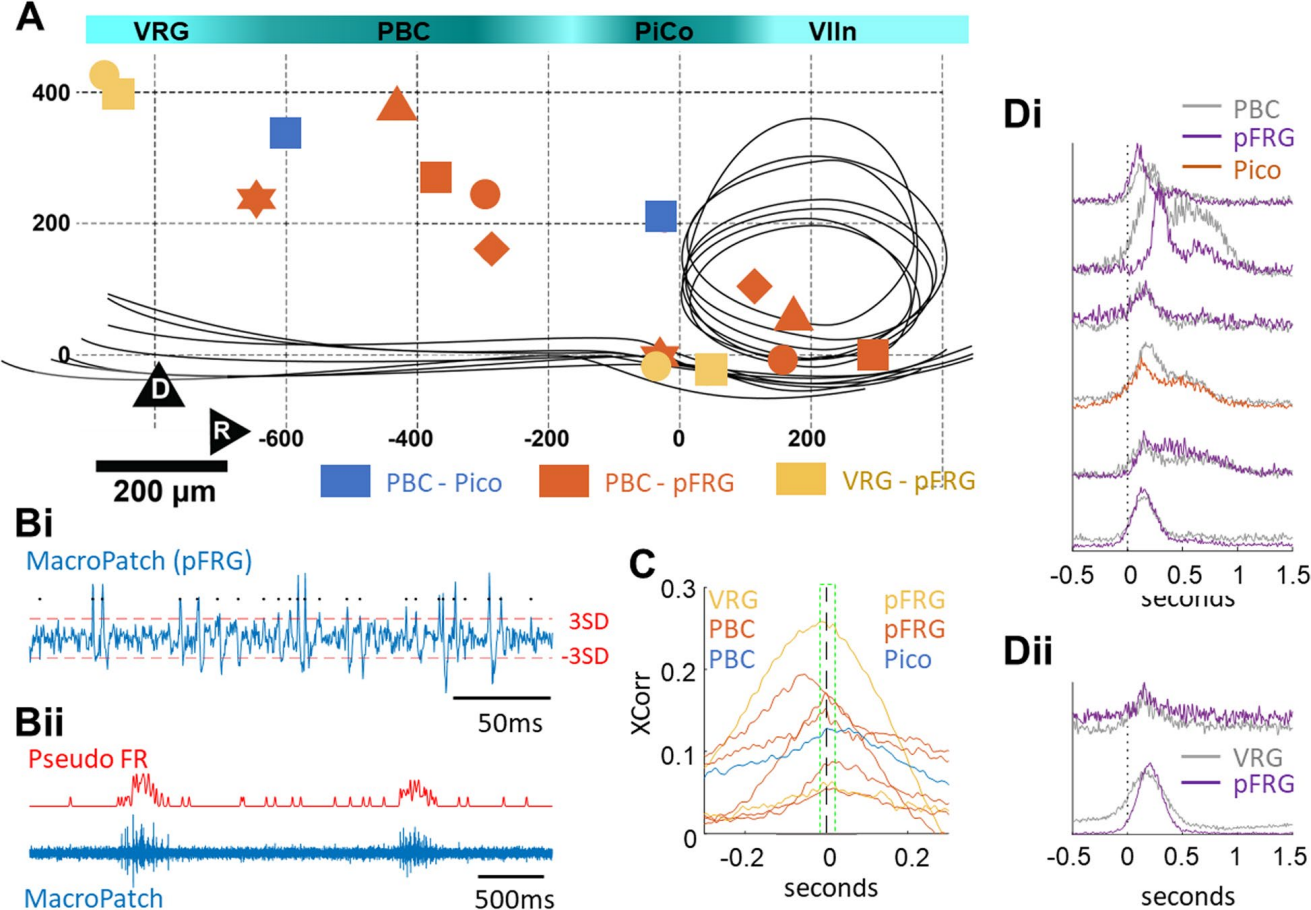
Macropatch recordings confirm tight synchronization of inspiratory onset along VRC networks. **A** Anatomical distribution of paired macropatch recordings from regions along the ventral respiratory column. Paired recordings of preBötC/pFRG (orange), VRG/pFRG (yellow), and preBotC/Pico (blue) were carried out in 7 preparations (indicated by distinct symbols).**B.i** Pseudo-spikes identified by thresholding the macropatch signal (criterion + -3 SD of the whole signal with a refractory period of 1.2 ms) are indicated by black points. **B.ii** Pseudo-spike times are convolved with a Gaussian window (SD = 3.7 ms, equivalent to a 270 Hz sampling rate) to obtain a pseudo-firing rate time signal which models a local summed potential of macropatch activity. **C** Cross-correlation of pseudo firing rate between paired recordings, whose anatomical sources are color-coded as in **A**. Peaks to the left of zero on the *x*-axis indicate that caudal structures led. Dashed green box spans ± 0.02 s. **D.i** We tested relative onset times by applying a threshold (set to 33% of the maximum pseudo firing rate for that recording) to detect burst onset using successively smaller Gaussian windows to ensure that threshold would not be trapped in a local minimum at the smallest 3.7 ms window. Both methods produced the same result: in preBötC/pFRG pairings, preBötC led pFRG in 2/5 experiments; in VRG/pFRG recordings, VRG led in 1/2 experiments, and in the PBC/PiCo pair onset times matched. In all cases but one, onset time differences led or lagged by less than 0.02 s (dashed green box, **C**). These findings are congruent with synchronous onset times detected using optical recording methods

The surprising observation that no anatomically compact region was identified as the source of inspiratory onset might have been due to the limited temporal resolution of optical recordings of slow Ca^2+^ transients [16]. To complement optical recordings, relative onset times along the VRC were estimated by positioning pairs of macropatch electrodes (*N* = 8 in 7 animals) to sample (20 kHz, resampled to 4 kHz) activity in or near VRG, preBötC, PiCo, and pFRG, identified from optical recordings (Fig. 3A). Inspiratory burst onset times were estimated by extracting pseudo-spikes (Fig. 3B.i, Additional file 4), which were convolved (SD 3.7 ms, resulting in an effective sampling rate of 270 Hz; Fig. 3B.ii), and then cross-correlated to detect differences in activation times between regions based in offsets from the midline of their cross-correlation (Fig. 3C). Tight synchronization was observed between preBötC and pFRG (4/5), VRG, and pFRG (2/2), as well as preBötC and Pico (1). In 2/3 of preBötC-pFRG pairs where differences in onset times would not have been resolvable using optical recordings, pFRG was found to lead. In the recording where preBötC activity clearly preceded pFRG, the offset of the cross-correlation peak would have been detected at optical recording sampling rates. Activity onset differences were also tested by averaging inspiratory bursts, aligned to burst onset (detected via thresholding at 33% of the maximum pseudospike firing rate). Results were qualitatively the same, with preBötC leading pFRG in 2/5 cases, and no discernible differences between PBC and PiCo or VRG and pFRG onset times were found (Fig. 3D.i, D.ii). Thus, signals obtained at high sampling rates did not reveal consistent differences in regional inspiratory onset times. Further, the observed variability in regional onset times matched what was observed in optical recording datasets.

### Neuronal variability is correlated with slow-timecourse variables

**Fig. 4:**
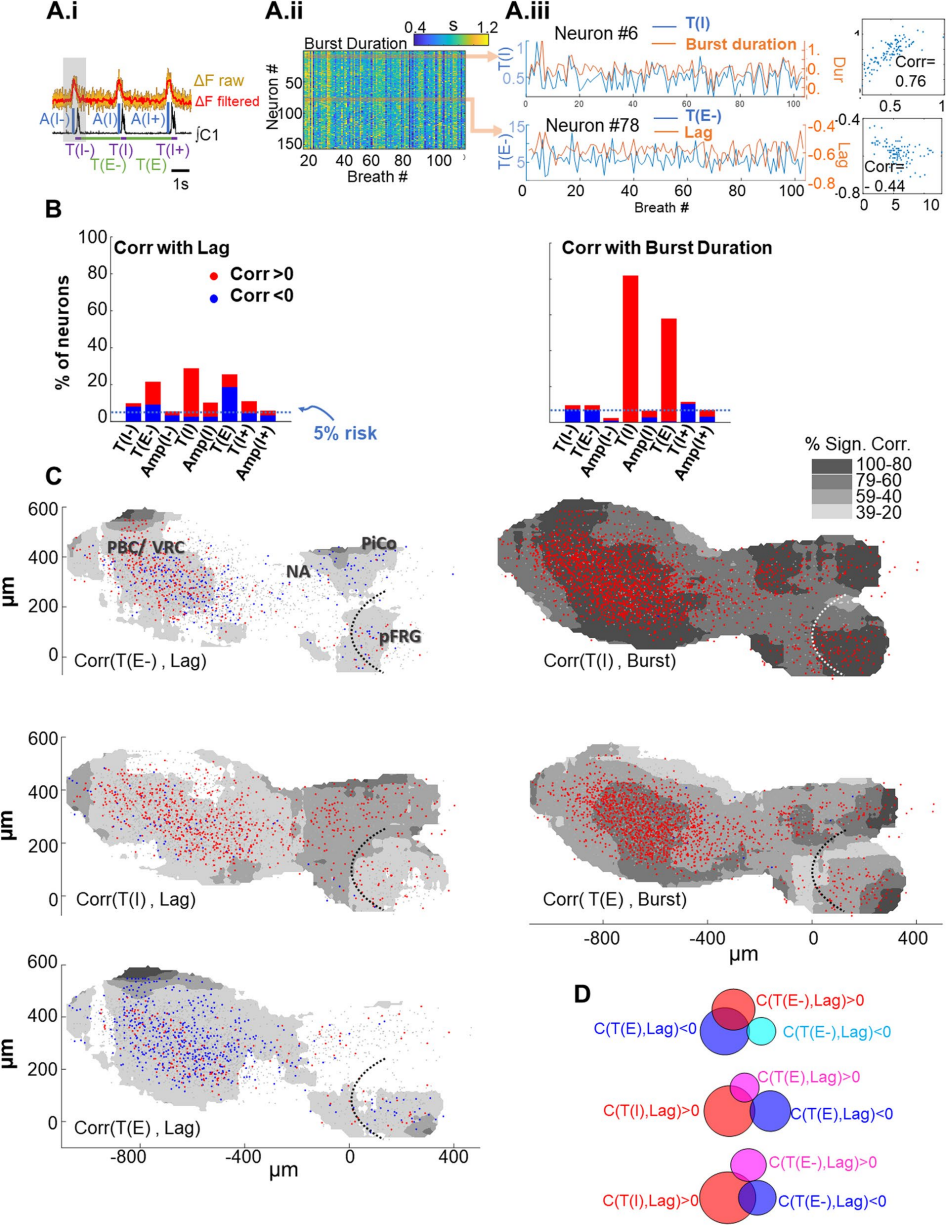
Variability of neuronal activity is correlated with motor output expiratory and inspiratory duration. **A.i** Definition of system-level variables correlated with lag and inspiratory burst duration of individual neurons. **A.ii** Heat map of burst durations from one optical recording. Over successive breaths, inspiratory duration of neuron 6 is positively correlated with motor output inspiratory duration (T(I)); similarly, the lag of neuron 78 is negatively correlated with the preceding expiratory duration (T(E-))(E-1) **A.iii** Top: time-series of neuron 7 burst duration (orange) and motor output (blue) inspiratory duration; when these variables are plotted against each other, strong positive correlation (0.75) is apparent (right). Bottom: time-series of neuron 78 lags (orange) and preceding expiratory duration (blue); when these variables are plotted against each other a weak negative correlation (0.44) is apparent (right). **B** Plot of the relative number of neurons with significant positive (red) and negative (blue) correlations between lag (left) and neuronal burst duration (right) and system-level variables shown in **A.i**. Dashed light-blue line indicates threshold below which correlations are expected by chance. Roughly 20% of neurons have lags that are significantly correlated with preceding expiratory duration (T(E-); positive), inspiratory duration (T(I); positive), and subsequent expiratory duration (T(E); positive and negative). More than 50% of neurons had burst durations that showed significant positive correlations with motor output T(I) duration and T(E). **C** Anatomical distribution of most robust correlations between neuronal lag (left) and neuronal burst duration (right) and system-level variables with positive (red) or negative (blue) correlations between lag and T(E-), T(I), and T(E). Gray shading represents the spatial average of the relative number of neurons positively or negatively correlated with each system-level variable. **D** Venn diagrams of neurons with significant positive and negative correlations between lag and system-level variables (T(E-), T(I), T(E))

In an earlier study, neuronal lags and burst durations were found to be correlated with motor output variables inspiratory duration (T(I), as well as preceding (T(E-)) and subsequent (T(E)) expiratory duration [8]. This was found to be the case here as well. As illustrated by data from one experiment, neuronal burst durations of individual neurons were positively correlated with (T(I) (Fig. 4A.iii, top), and neuronal lags were correlated with T(E) (Fig. 4A.iii, bottom). In pooled data, significant positive and negative correlations were found (*α* > 5%; Fig. 4B) between lags (Fig. 4B, left) and T(E-), T((E), and T(I); burst durations (Fig. 4B, right) were significantly correlated with T(I) and T(E). Neurons whose lag and/or burst durations were significantly correlated with T(E-), T(E) and T(I), were distributed along the VRC in overlapping large clusters (Fig. 0.4C). Interestingly, subsets of neurons were correlated with more than one system-level variable; thus, 22% of neurons whose lag was negatively correlated with subsequent expiratory duration were also positively correlated with previous expiratory duration (Fig. 4D). Thus, networks that are modulated by (or regulate) slow-timecourse variables show substantial anatomical overlap and are distributed along the VRC. This anatomical dispersion is also seen in neurons activated earliest in the cycle (Fig. 2C.i, Video in Additional file 2). While these maps overlap, their distributions differ: neurons strongly correlated with T(E-) and T(E) are densest in preBötC (S4 C), while the proportion of earliest inspiratory neurons was highest in pFRG/RTN (Fig. 2C.ii).

### Synaptic blockade reveals syncytial oscillations along the VRC

Endogenous bursters have been proposed as essential constituents of rhythmogenic networks in both preBötC [17] and pFRG/RTN [18]. To assess whether they might coordinate the observed distributed initiation of inspiratory bursts, a broad-spectrum synaptic blocker cocktail was applied, silencing respiratory motor output within 2 min of drug application. Patency of blockade was validated by showing that following synaptic blockade, post-synaptic networks mediating swallow were no longer activated by stimulation of putative command networks for swallow in dorsomedial medulla [19] (Video in Additional file 5).

**Fig. 5:**
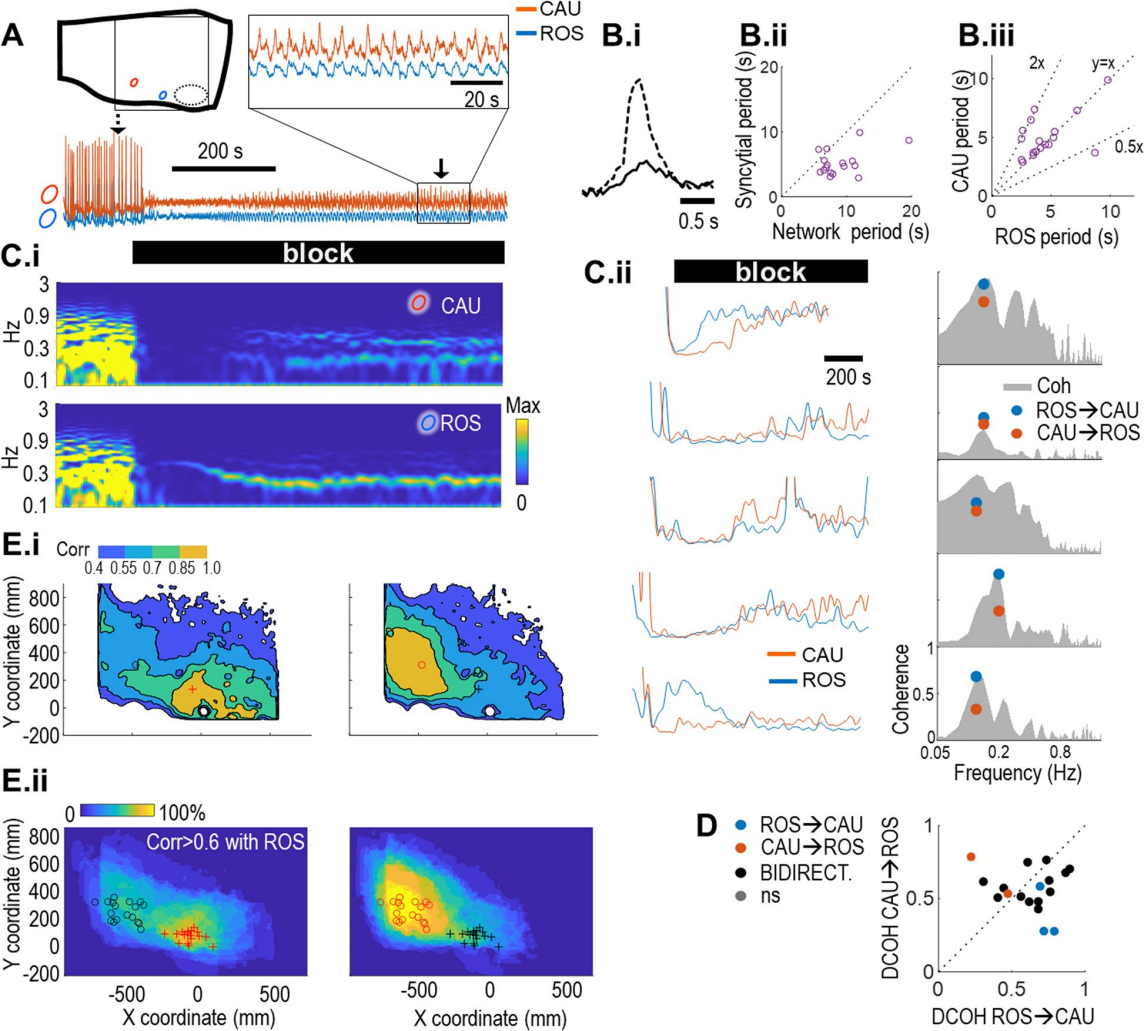
Following synaptic blockade, synchronized stationary rhythmic activity emerges along the VRC. **A** Top left: cartoon of a view of the sagittal face of the preparation with a typical recording location superimposed. Orange and blue circles represent the approximate locations of the field potential ROIs used in these experiments, black dashed oval: VIIn. Bottom: optical recordings were made from large “field potential” ROIs at a caudal location (CAU) overlapping with preBötC (orange traces throughout), and at a rostral location (ROS) overlapping with pFRG/RTN (blue traces throughout). Recordings made before and during blocker cocktail application (black bar) reveal the transition from quiescence to fast, stationary, low amplitude rhythmic activity along the VRC. Top right: expanded view of traces outlined in the box below **B**. **B.i** Peaks before (broken line) and after (solid line) synaptic blockade, averaged over both ROIs in individual bursts indicated by arrows in **A**. **B.ii** A plot of periods in the intact network against syncytial periods reveals lower variability in syncytial periods. **B.iii** A plot of rostral periods against caudal periods reveals that, while in most experiments rostral and caudal periods matched, caudal periods were often twice the rostral period. **C.i** Spectrograms of frequencies between 0.1 and 0.3 Hz before and during blocker cocktail application caudally (top) and rostrally (bottom) for the experiment shown in **A**. **C.ii** left: traces of maximum power between 0.1 and 0.3 Hz during cocktail wash-in from caudal (orange) and rostral (blue) ROIs for 5 experiments reveal that oscillations start simultaneously in both locations or earliest rostrally; right: coherence (gray) between the two signals in the time interval 700–1300 s after emergence of syncytial rhythm. Directed coherence values taken at the coherence peak (blue and orange points) reveal strong bidirectional or rostral to caudal couplings. **D** DCOH at the coherence peak for pooled experiments. All DCOH values are significant in at least one coupling direction. **E.i** Cross-correlations of a reference pixel and all other pixels—obtained using the pixel at the center of mass of the rostral (left; cross) and caudal (right; circle) ROIs delineates the region of synchronous syncytial rhythm in one experiment. **E.ii** Pooled correlations between pixels from 18 experiments define a region extending rostrally from preBötC and caudally from pFRG/RTN in the intact network

At lower sampling rates selected to enhance detection of weak signals, we recorded activity along the entire VRC. Surprisingly, while neurons with stationary pacemaker activity were rare, we detected diffuse, low-amplitude synchronized rhythmic activity along the VRC that emerged after a transitional quiescent interval (Fig. 5A; Video in Additional file 6; dataset in Additional file 7). The expanded view of 100 s of activity (Fig. 5A, box) illustrates the stationary and phase-locked oscillations typically found across experiments. Signals were lower in amplitude, and the steep rise and exponential fall typically seen during inspiratory bursts under baseline conditions was replaced by symmetric peaks (Fig. 5B.i), consistent with the profile of burstlets proposed by others to have rhythmogenic function [20]. Steady-state syncytial rhythm was faster than respiratory rhythm in the intact network and less variable (Fig. 5B.ii). Rhythmic activity at the level of the rostral ROI typically had a 1:1 or 2:1 rhythm in relation to the caudal ROI (Fig. 5B.iii, *N* = 18).

A spectrogram of signals recorded at rostral and caudal ROIs revealed that the syncytial rhythm typically emerged first rostrally and remained quasi stationary over more than 10 min (Fig. 5C.i. *N* = 6). Across experiments, syncytial rhythm initiated simultaneously or rostrally (Fig. 5C.ii, left). This was confirmed by the calculation of directed coherence (DCOH) [21] taken at the frequency of the classical coherence peak (Fig. 5C.ii, right). We found similar results in the *N* = 18 other experiments recorded following emergence of stationary activity (Fig. 5D): all couplings were significant and most were bidirectional. Bidirectional coupling reveals strong interactions between caudal and rostral syncytial rhythms; this also suggests that syncytial oscillations are not initiated in a particular region. To provide a more graded anatomical description of the syncytium, cross-correlations of a reference pixel and all other pixels were computed, using the center of mass of rostral (Fig. 5E.i left; cross) and caudal (Fig. 5E.i right; circle) ROIs, shown for one experiment and for pooled experiments (*N* = 18) (Fig. 5E.ii). These maps reveal regions that extend rostrally from the caudal region and caudally from the rostral region with some overlap. These regions together overlap with maps of respiratory network constituents (Fig. 1C).

### Gap-junction blockers silence respiratory and syncytial rhythm

**Fig. 6:**
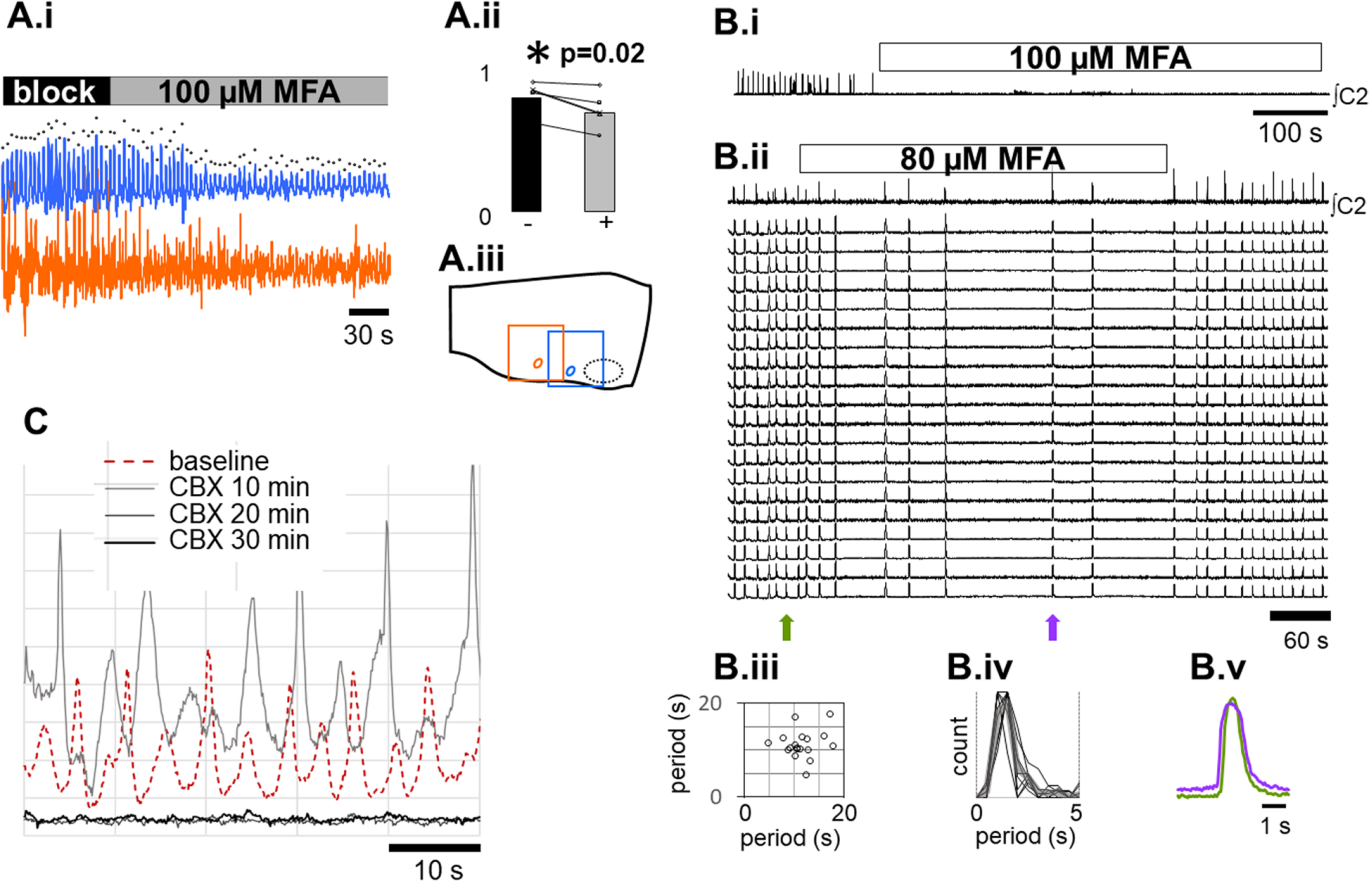
Gap-junction blockers disrupt fictive respiration in the intact network and attenuate or silence syncytial oscillations. **A.i** Syncytial activity recorded rostrally (blue) and caudally (orange) reveals rapid attenuation of oscillation amplitude following application of 100 μM gap-junction blocker meclofenamic acid (MFA) under synaptic blockade; points in the upper trace mark the peaks used to quantify amplitudes. **A.ii** MFA significantly reduces oscillation amplitude (paired *t*-test, *p* = 0.02). **A.iii** Cartoon indicating the field of view of rostral and caudal 20X recordings of syncytial activity. **B.i** In the intact network, bath application of 100 μM MFA rapidly silences respiratory motor output (top trace) and network activity (not shown). **B.ii** At 80 μM, MFA slows motor output (top trace), as well as network activity (lower traces), but no neurons active in the intact network fall silent nor are ectopic bursts over subnetworks detected. **B.iii** Poincaré plot of periods from the dataset shown in B.i do not display discrete clusters, as would be expected if drive to (pre)motoneurons was disrupted, leading to periods at integer multiples of control [26]. **B.iv** Histogram of post-MFA wash-in periods divided by control periods (*N* = 7) forms a skewed unimodal distribution. If MFA disrupted transmission of rhythmic drive to (pre-)motoneurons, we would expect to see multiple peaks; their absence suggests that gap junctions play a role in stabilizing respiratory period. **B.v** Averaged burst amplitude for 1 cycle before (green arrow in **B.ii**, green trace) and during MFA wash-in (purple arrow in **B.ii**, purple trace) reveals that neither inspiratory burst amplitude nor onset were attenuated by MFA. **C** Application of the broad-spectrum gap-junction blocker carbenoxolone (CBX, 100 μM; *N* = 6) transiently increased syncytial amplitude (10 min, gray line) compared to baseline (red line); thereafter, frequency slowed, and amplitude was attenuated (20 min, fine black line), and after 30 min, it was undetectable (30 min, thick black line)

The diffuse, tightly synchronized, low-amplitude rhythm observed under synaptic blockade is consistent with oscillatory dynamics of a gap-junction-coupled syncytium [22]. To test this conjecture, we applied meclofenamic acid (MFA; 60 μM–100 μM), which blocks both gap junction protein connexin 43 (CX43) [23] and connexin 36 (CX36) [24], which are expressed in neurons and glia during mouse development [25][25]. In these experiments (*N* = 5), 20X recordings localized to either caudal or rostral regions (Fig. 6A.i) were made under synaptic blockade before and during wash-in of MFA.

MFA significantly attenuated syncytial peak amplitude (*p* = 0.02; Fig. 6A.i, A.ii). In the intact network (*N* = 7), 100 μM MFA silenced respiratory motor output (Fig. 6B.i). At lower concentrations in the intact network (40–80 μM; *N* = 7), MFA slowed but did not stop respiratory rhythm (Fig. 6B.ii). The lack of clustering at integer multiples of control period in Poincaré plots of respiratory periods from an individual experiment (Fig. 6B.iii) suggests that MFA-induced slowing arose from disruption of rhythmogenic processes rather than disruption of respiratory drive [26]. This was corroborated by the left-skewed unimodal distribution in histograms of normalized periods (Fig. 6B.iv), which would have been multimodal if slowing was due to skipped cycles. The findings that MFA attenuates syncytial rhythm under synaptic blockade and slows respiratory period in the intact network provides support for the conjecture that syncytial oscillations are in part mediated by CX36 and/or CX43 gap junctions and stabilize respiratory rhythm.

While respiratory periods lengthened at lower MFA concentrations, all neurons that were active under baseline conditions remained active and tightly synchronized following MFA wash-in (Fig. 6B.ii), and burst profile and amplitude were also unaffected (Fig. 6B.v). These findings are consistent with findings by other groups that inspiratory burst amplitude is regulated independently from rhythmogenesis [27].

To rule out the possibility that the observed attenuation was due to non-specific effects of MFA and to assess whether the residual syncytial oscillations that persisted following MFA wash-in might be due to gap junctions other than CX-43 and CX-36, after blocking synaptic transmission and detecting syncytial oscillations, we applied the broader spectrum gap-junction blocker carbenoxolone (CBX, 100 μM; *N* = 6). After 10 min, syncytial amplitude increased, and frequency slowed at 20 min oscillation amplitude was strongly attenuated, and after 30 min was undetectable (Fig. 6C).

### Diffuse synchronous rhythmic activity along the VRC is mediated by both neurons and glia

**Fig. 7:**
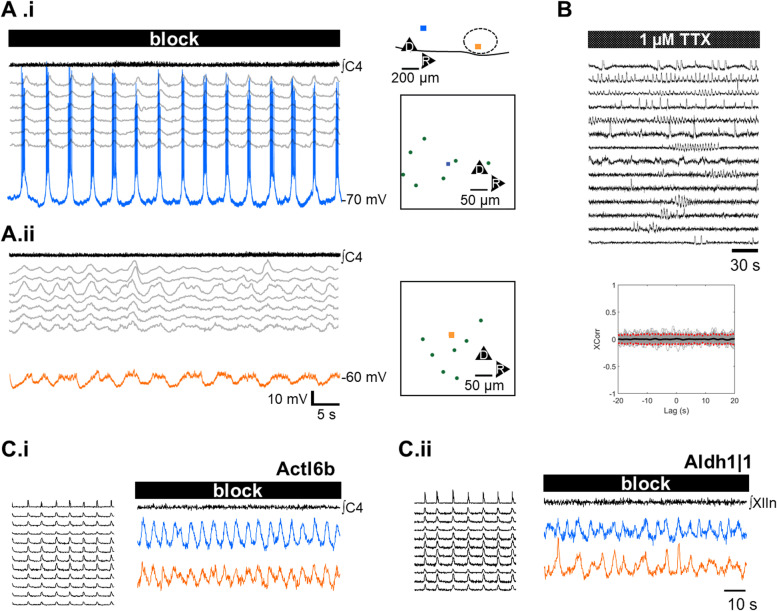
Both neurons and glia are constituents of the syncytium. **A** To directly test whether neurons participated in syncytial oscillations, patch-clamp recordings were carried out following synaptic blockade (*N* = 4). Here, serial patch clamp recordings in the same preparation following synaptic blockade at the level of pFRG (blue trace and square, A.i) and preBötC (orange trace and square, A.ii) are shown. Intracellular recording locations relative to VIIn (dashed oval) reconstructed from images obtained during optical recording are shown in cartoon at top right. Fluctuations in membrane potential phase-locked to syncytial activity detected at ROIs obtained during concurrent optical recording (gray traces) confirms that neurons participate in syncytial activity. Boxes to the right of traces indicate the location of ROIs (green dots) in relation to the patch electrode tips (orange and blue squares), establishes that syncytial rhythm could be detected far from the patch-clamped cell, consistent with gap-junction coupling. **B** Following bath application of the Na_(V)_ blocker TTX (1 μM), stationary syncytial rhythmic activity was replaced by transient spindle activity (top), which was uncoupled from neighboring activity, as confirmed by the absence of peaks in cross-correlations between all traces (bottom). **C** To directly assess the contribution of neurons and glia to syncytial oscillations, we crossed GCaMP6F mice with mice expressing cre- in neurons (Tg(Actl6b-Cre)4092Jiwu/J) and glia (B6;FVB-Tg(Aldh1l1-cre)JD1884Htz/J) respectively. Prior to synaptic blockade, both mice expressing GCaMP in neurons (**C.i**, left panel; *N* = 3) and glia (**C.ii**, left panel, *N* = 5) showed robust activity during inspiration. Following synaptic blockade, fluctuations in luminance matching syncytial rhythm in germline mice was detected (**C.i**, **C.ii**, right panels). Taken together, these findings confirm that neurons participate in syncytial oscillations and suggest that glia do so as well

To directly confirm that syncytial oscillations reflected fluctuations in neuronal membrane potential, patch clamp recordings were carried out. As predicted by optical recording data, neurons were found to display oscillations in membrane potential synchronous with syncytial oscillations detected in surrounding neuropil via concurrent optical recordings. We illustrate these findings from one experiment, in which we sequentially patched onto a neuron in pFRG (shown in blue, Fig. 7A.i) and preBötC (shown in orange Fig. 7A.ii). Patch electrode recording locations are indicated in schematic in upper right Fig. 7A.i, and traces from concurrent optical recordings are shown in gray, with ROI locations in relation to the patch electrode tip shown in schematics to the right of the traces. (Fig. 7A.i, 7A.ii).

Recent evidence suggests that rhythmic motor patterns may arise out of the dynamics of a glial syncytium [22]. To characterize the role of glia in the propagation of network-wide rhythmic activity, we applied the broad-spectrum Na_(V)_ blocker tetrodotoxin (1 μM; *N* = 4), which selectively silences neuronal activity. Following wash-in, motor output immediately ceased, and synchronous rhythmicity along the VRC was replaced by uncoupled spindle-like activity from spatially compact ROIs; (Fig. 7B, top); synchronized anatomically dispersed stationary rhythmic activity was abolished, as confirmed by the lack of peaks in the cross-correlation of spindle activity (Fig. 7B bottom). These observations suggest that neurons are necessary for generating stationary syncytial oscillations along the VRC.

To more directly test whether glia contribute to the observed syncytial oscillations, we biased GCaMP6F toward glial expression in the inducible cre- dbx1 mouse by tamoxifen exposure after the peak neuronal birth period (E10-E12) [28]. We recorded optically from inducible-cre Dbx1-GCaMP6F mice exposed to tamoxifen at E12.5 (*N* = 2) and E13.5 (*N* = 3) and used Dbx1-GCaMP6F mice exposed to tamoxifen at E9.5 (*N* = 6) as controls. In mice injected at E9.5, strong activity phase-locked with respiratory motor output originating from a region overlapping with preBötC was detected (Additional file 8 A, left). And syncytial rhythm persisted following synaptic blockade (Additional file 8 A, right), consistent with the conjecture that syncytial oscillations are mediated by neurons. In mice injected at E12.5-E13.5, optical recordings under baseline conditions revealed oscillations phase-locked to motor output in preBötC (Additional file 8 B left). Following synaptic blockade, rhythmic activity in preBötC ceased shortly after motor output was silenced (Additional file 8 B, right).

To more robustly confirm the contribution of glia or neurons to syncytial oscillations, we selectively expressed GCaMP6F in neurons by carrying out experiments on first-generation heterozygous mice obtained by crossing the Tg(Actl6b-Cre)4092Jiwu/J mouse, which has pan-neuronal cre expression, with the B6J.Cg-Gt(ROSA)26Sortm95.1(CAG-GCaMP6f)Hze/MwarJ mouse; similarly, we studied first-generation heterozygous mice obtained by crossing the B6;FVB-Tg(Aldh1l1-cre)JD1884Htz/J mouse, which expresses cre predominantly in astrocytes and oligodendrocytes, but also very limited expression in neurons [29], with the B6J.Cg-Gt(ROSA)26Sortm95.1(CAG-GCaMP6f)Hze/MwarJ mouse. Consistent with what we observed in the inducible cre- dbx1 mice injected at E9.5, syncytial oscillations were detected in recordings from mice expressing GCaMP6F in neurons, providing further support for the conjecture that neurons participated in syncytial dynamics (Fig. 7C). Conversely, in mice that predominantly targeted astrocytes and oligodendrocytes with GCaMP6F expression, we also observed syncytial oscillations following synaptic blockade (Fig. 7D). Differences in outcomes between differing strategies to target glia selectively for optical recording may be due to GCaMP6F expression in glial cells not under the regulation of dbx1, or to unwanted GCaMP6F expression in a fraction of the neurons.

## Discussion

Mice expressing GCaMP6F in the germline were used to optically record phenotypically heterogeneous respiration-modulated networks along the VRC in ventrolateral medulla, using the sagittally sectioned rodent hindbrain preparation. This approach enabled long recording epochs from all respiration-modulated cells at or near the surface of the preparation, at sampling rates high enough to capture inspiratory onset sequences across preBötC, pFRG/RTN, and PiCo. We used this platform to investigate the locus of inspiratory burst initiation and coupling between networks along the VRC.

A robust finding across experiments was that ROIs active earliest during inspiratory onset were distributed along the VRC, with the highest relative numbers in pFRG/RTN; most onset sequences conformed with the median onset sequence, but atypical sequences were observed in all experiments; and overlapping, anatomically dispersed ROIs had onset times and burst durations that were significantly correlated with system-level variables T(I), T(E-) AND T(E). In a subsequent set of experiments, syncytial oscillations spanning the VRC were observed following synaptic blockade. These oscillations first emerged rostrally, were faster and less variable across preparations than respiratory rhythm in the intact network in that same cohort, and showed strong, predominantly bidirectional coupling between rostral and caudal regions. Cross-correlations between reference pixels from rostral and caudal ROIs, and all other pixels provided an estimate of the anatomical extent of syncytial oscillations, revealing that they spanned the VRC, from a region caudal to preBötC, extending rostrally along the ventral margin of the VRC, to a region overlapping with pFRG/RTN. Gap junction blockade significantly reduced syncytial oscillation amplitude in synaptically isolated networks and silenced or slowed respiratory rhythm in the intact network. Using distinct mouse lines to specifically target GCaMP6F to neurons and glia revealed that syncytial oscillations could be detected in both types of cells. Because syncytial oscillations were eliminated under TTX, we infer that neurons are necessary for syncytial oscillations.

The use of widefield microscopy to record Ca^2+^ dynamics using mice expressing GCaMP6F in the germline provides inclusive access to mesoscopic dynamics along the VRC, but the limitations and trade-offs associated with these methods must be factored into interpretation of the data. First, because all cells in these mice express GCaMP6F, activity of neurons and glia are conflated. Second, signals detected within an ROI at the surface of the slice may emanate from multiple vertically aligned signal sources. Third, recordings were carried out at timescales over an order of magnitude slower than synaptic transmission, from an indicator whose slow kinetics precludes higher temporal resolution [16].

To overcome the conflation of glial and neuronal signals, we have carried out a small number of experiments in mice expressing GCaMP6F in neurons only, and glia predominantly, establishing that both inspiratory bursts and syncytial oscillations were detectable in neurons and glia. Thus, we were able to disambiguate findings obtained in germline-GCaMP6F mice. Further, to confirm participation of neurons in syncytial dynamics, a small number of current-clamp recordings from single neurons were carried out following synaptic blockade. These established that neuronal membrane potential displayed oscillatory fluctuations in membrane potential that matched syncytial oscillations. Optical recordings carried out in parallel detected syncytial oscillations dispersed across the field of view that were synchronous with the oscillations recorded in the single neuron, supporting the conjecture that syncytial oscillations were mediated by gap-junction coupling between respiratory network constituents, consistent with what others have found [30]. This conjecture found independent support in the observation that gap-junction blockers attenuated or eliminated syncytial oscillations.

Although 2-photon microscopy would eliminate the one-to-many mapping between ROIs and signal sources, thereby giving access to a finer-grained description of dynamics along the VRC, it is not clear that the findings of this study are weakened by the possible conflation of signals from multiple vertically aligned neurons. The observation that inspiratory onset in the synaptically intact network occurred synchronously along the VRC holds even if signals recorded along the column are generated by cells overlapping in the z-plane. The number of atypical onset sequences may have been underestimated because of the possibility of more than one neuron contributing to signals recorded at a given ROI, but the atypical onset sequences that were detected are unaffected by limitations in resolving activity from single neurons, since atypical onset sequences are identified based on activity detected at anatomically distinct ROIs; finally, because the fluctuations in luminance associated with syncytial activity were inherently diffuse across the neuropil, mappings onto somatic sources are ill-defined.

To bridge the gap between the timescale of synaptic transmission, and the low-pass filtered Ca^2+^ dynamics measured here as a surrogate for neuronal activation, we carried out paired macro-patch recordings along the VRC, enabling voltage recordings from groups of neurons at high sampling rates. This approach avoided the sampling artifact that would have occurred with paired single-unit recordings, since given the biophysical heterogeneity of neurons along the VRC, the finding that in any given pair of neurons a particular distribution of burst onset times was observed would provide little insight into distribution of onset times across the network. Sampling from a population of neurons via macropatch also imposes limitations: the mapping between activity in individual neurons and the pseudo-spikes detected here via thresholding is unclear, and the detection of burst onsets requires temporal smoothing, which ultimately reduced the temporal resolution of this approach to 270 Hz. Nonetheless, the observation that in almost all macropatch electrode pairs distributed along the VRC onset times were synchronous provides support for the matching observation obtained via optical recording.

Some of our findings are congruent with earlier studies. Consistent with the conjecture that the preBötC contains networks essential for respiratory rhythmogenesis, we find that the region identified in optical recordings from mice in which preBötC neurons selectively express GCaMP6F overlaps with the region in which the largest number of respiration-modulated ROIs were found in germline-GCaMP6F mice and also overlaps with somatostatin receptor expression, as reported by others [31, 32], confirming the anatomical boundaries of preBötC. Similarly, respiration-modulated neurons at the ventral margin of VIIn overlap with phox2b^+^ neurons, consistent with earlier reports on the pFRG/RTN [33]; in addition, respiration-modulated neurons dorsal and medial to VIIn overlap anatomically with the lateral edge of PiCo [10]. Finally, the syncytial oscillations observed here following synaptic blockade are qualitatively similar to the burstlet rhythm in the transverse slice [11], which has been proposed by others to generate respiratory rhythm [5, 34].

Conversely, the finding that neurons with tightly synchronized onsets were anatomically dispersed along the VRC appears incompatible with the hypothesis that respiratory rhythm is generated by a spatially compact network within the preBötC [5, 35, 36]. Further, we find that a larger proportion of neurons at or near the pFRG/RTN are active earliest during inspiration, and that syncytial oscillations emerge first at the level of pFRG/RTN following synaptic blockade. These observations suggest that the the pFRG/RTN at least contributes to respiratory rhythmogenesis in neonate mice and are consistent with an alternative hypothesis that pFRG/RTN networks are essential for respiratory rhythmogenesis [9]. These inferences must be treated with caution. If the pFRG/RTN were the locus for respiratory rhythm generation, then respiratory rhythm would be expected to initiate from this region in every cycle. In addition, removal of this structure should eliminate respiratory rhythm; this is clearly not the case [4, 37]. In addition, the earliest emergence of syncytial oscillations at the level of the pFRG/RTN does not imply that the syncytial rhythm is generated by these networks, particularly because once established this activity persists with strong bidirectional coupling. Lastly, the oscillatory dynamics along the VRC following synaptic blockade does not necessarily imply that syncytial oscillations play a causal role in respiratory rhythmogenesis.

Despite these caveats, the evidence obtained here of tight synchronization in burst onsets along the VRC under baseline conditions, together with the observation of synchronous oscillations across a syncytium spanning the VRC following synaptic blockade is congruent with the claim that gap junctions synchronize activity over spatially dispersed networks [38]. More speculatively, the observation that at low concentrations, MFA disrupts slow-timescale regulation of respiratory period without impacting fast-timescale synchronization of the inspiratory bursts suggests a possible role of the syncytium in the regulation of respiratory rhythm. On this view, synaptic transmission would regulate the fast dynamics of the system while electrotonic coupling would regulate slow dynamics. This partitioning between fast and slow processes is a ubiquitous feature of mathematical models of respiratory rhythmogenesis [39–41]. Although an account of how electrotonic coupling might regulate respiratory period is beyond the scope of this study, others have proposed models of network rhythmogenesis at a similar frequency to fictive respiration, mediated by electrotonic coupling between biophysically heterogeneous non-bursting neurons in the inferior olive [42], a circuit overlapping with networks described here. At the least, if electrotonic coupling between neurons is a property of VRC networks, this finding may constrain the set of models that can qualitatively reproduce features of respiratory rhythm generation and may provide an account for correlations detected here between ROI burst onsets and durations and subsequent or preceding periods.

The detection of neuronal electrotonic coupling along the VRC may be a transient feature apparent in the immediate postnatal period, when oligosynaptic transmission between cells far apart occurs in incompletely myelinated networks [43]. Recent findings suggest that gap junctions transiently expressed in early postnatal development contribute to respiratory patency in neonates: Cx36 knockout mice displayed irregular respiratory patterns and were labile to hypoxic and hypercapnic challenge, consistent with morbidities associated with sudden infant death syndrome [44]. Thus, syncytial coupling along the VRC may only play a role in early development.

The persistence of gap junctions in adults would extend the functional repertoire of VRC networks however. As elegantly delineated in an analysis of the effect of gap junctions on network dynamics in the crustacean stomatogastric CPG [45], the overlay of electrotonic and synaptic coupling enables both lability and robustness: multiple combinations of synaptic and gap junction-mediated coupling can give rise to near-identical rhythmic activity patterns or, conversely, small alterations in gap junction coupling strength produced qualitatively different dynamics between network constituents, consistent with degeneracy [46, 47]. Thus, in the presence of gap junctions, multiple trajectories to stable rhythmic motor output may coexist. Such processes would account for the coexistence of typical and atypical onset sequences observed in all high-speed optical recordings in the intact network, and weaken inferences of coupling obtained using time-series methods, which assume stationarity [8].

The ambiguity that gap junctions impose on observations that would otherwise support mechanistic inferences about network function may reflect their utility. In the simpler model system referenced above, gap junctions both expand the set of stable regimes the network can converge towards and expand the set of network configurations that give rise to a particular regime. This suggests that the presence of gap junctions enables the multiplexing of a range of behaviors and regulatory functions inherent to respiratory networks [48]. These include blood-gas homeostasis, the intercalation of diverse orofacial behaviors within the respiratory cycle, and the poorly understood developmental demands of maintaining basic function as respiratory networks undergo profound transformation during a critical period starting at P11 in mice [49], among others. In the context of the heterogeneity and complexity of tasks that these networks must simultaneously accomplish, the long-held aim of identifying the minimal network necessary and sufficient for respiratory rhythmogenesis may be ill-defined [50].

## Methods

### Mouse lines

In accordance with methods approved by the Institutional Animal Care and Use Committee of the University of Louisville, we carried out studies in the following mouse lines: B6.Cg-Tg(Camk2a-cre)T29-1Stl/J and B6.Cg-Edil3Tg(Sox2-cre)1Amc/J mice [51], B6.Cg-Edil3Tg(Sox2-cre)1Amc/J mice [52], Tg(Actl6b-Cre)4092Jiwu/J mice [53], B6;FVB-Tg(Aldh1l1-cre)JD1884Htz/J mice [54], and B6J.Cg-Gt(ROSA)26Sortm95.1(CAG-GCaMP6f)Hze/MwarJ mice [55], all obtained from Jackson Laboratories (Farmington CT). In addition, mice expressing a tamoxifen-sensitive Cre recombinase (Dbx1CreERT2; CD1 background strain) [56] were kindly provided by Dr. Christopher del Negro. Genotypes were verified by PCR using genomic DNA isolated from mouse tail biopsies.

Because Tg(Camk2a-cre)T29-1Stl express Cre recombinase expression in several structures of the male germline, when male mice are bred with floxed females, spontaneous germline deletion of the floxed allele occurs in 25% offspring [12]. This pattern of breeding was exploited to obtain mice expressing GCaMP6F in the germline. To ensure reproducibility, germline GCaMP6F was replicated using a different mouse line by crossing female B6.Cg-Edil3Tg(Sox2-cre)1Amc/J mice, which express Cre recombinase in epiblast cells, with B6J.Cg-Gt(ROSA)26Sortm95.1(CAG-GCaMP6f)Hze/MwarJ mice to more easily obtain germline-GCaMP6F expression. In both strains, germline expression was verified by establishing ubiquity of expression, confirmed by staining for GFP in heart. In order to assess the contributions of neurons and glia to network activity both under baseline conditions and following synaptic blockade, two approaches were taken. First, we bred double-homozygous B6J.Cg-Gt(ROSA)26Sortm95.1(CAG-GCaMP6f)Hze/MwarJ—Dbx1CreERT2. A female and male mouse from this colony were housed together overnight; the female was then weighed and housed separately and weighed again 1 week later. Mice that had gained 2 g or more in weight were assumed to be pregnant and were injected with tamoxifen dissolved in corn oil (10 mg/ml) in volumes proportional to the mouse’s weight (60 μL–90 μL for mice ranging in weight between 20 and 35 g) at either E9.5 (to bias towards GCaMP6F in neurons) or E12.5-E13.5 (to bias towards GCaMP6F in glia) [28]. At E19.5, pups were removed from the dam via cesarean section and placed with a surrogate dam, before being harvested for recording at P0-P5. To corroborate findings obtained using inducible cre- mice, we carried out experiments on first-generation heterozygous mice obtained by crossing B6J.Cg-Gt(ROSA)26Sortm95.1(CAG-GCaMP6f)Hze/MwarJ mice with Tg(Actl6b-Cre)4092Jiwu/J founder mice, to generate mice with pan-neuronal GCaMP6F expression [53]. Similarly, we carried out experiments on first-generation heterozygous mice obtained by crossing B6J.Cg-Gt(ROSA)26Sortm95.1(CAG-GCaMP6f)Hze/MwarJ mice with B6;FVB-Tg(Aldh1l1-cre)JD1884Htz/J mice to generate mice with GCaMP6F expression predominantly in astrocytes and oligodendrocytes [54],

### Surgery

Germline-GCaMP6F pups (P0-P6) were anesthetized with isoflurane; the forebrain was exposed and removed, enabling isolation of the hindbrain and spinal cord in chilled artificial cerebrospinal fluid (aCSF) made up of (in mM) 128.0 NaCl, 3.0 KCl, 1.2 CaCl_2_, 1.0 MgSO_4_, 21.0 NaHCO_3_, 0.5 NaH_2_PO_4_, and 30.0 glucose, equilibrated with 95% O_2_–5% CO_2_. The hindbrain was transected at the level of the sensory portion of the trigeminal nerve, just rostral to the facial nucleus (VIIn). The preparation was then mounted with the dorsal side down on an attachment permitting sectioning at compound angles [57], using the basilar artery to reproducibly align the preparation along the midline of the chuck. A sagittal section was cut at the lateral edge of the VIIn, visible at the surface of the preparation, at 17.7° ventrodorsal tilt and 3.7° rostrocaudal tilt relative to the midline, to expose the VRC along its major axis. The preparation was mounted on the face of a Sylgard (Dow Corning, Midland MI) block cut at the ventrodorsal tilt angle, so that the sagittal face of the preparation, aligned with the top edge of the Sylgard block, was perpendicular to the light path. A razor-blade fragment embedded in the Sylgard block enabled the preparation to be stabilized in the recording chamber by the placement of a 10-mm neodymium magnet on the undersurface of the recording chamber.

### Pharmacology

To synaptically isolate respiratory networks, a cocktail was used consisting of AMPA receptor blocker 2,3-dioxo-6-nitro-1,2,3,4-tetrahydrobenzo[f]quinoxaline-7-sulfonamide disodium salt (NBQX; 10 μM), NMDA receptor blocker D-(-)-2-amino-5-phosphonopentanoic acid (D-AP5; 50 μM), GABA_(A)_ receptor blocker bicuculline methobromide (10 μM), group 2 MGluR blocker LY 341,495 (500 nM), GABA_(B)_ receptor blocker CGP 52,432 (10 μM) (all from Abcam, Waltham MA), and glycine receptor blocker strychnine (2 μM; Sigma-Aldrich, St Louis MO). To block gap junctions, the selective, reversible CX-43 and CX-36 channel blocker meclofenamic acid sodium (MFA; 60–100 μM; Sigma-Aldrich, St Louis MO) was applied alone or in conjunction with the blocker cocktail. To confirm results obtained using MFA, the broad-spectrum gap junction blocker carbenoxolone (CBX; 100 μM; Sigma-Aldrich, St Louis MO) was applied following synaptic blockade. NA^+^_(V)_ conductances were blocked with tetrodotoxin (TTX; 1 μM; Sigma-Aldrich, St Louis MO). All solutions were prepared the day of the experiment and recirculated to enable longer recordings with low solution volumes.

### Voltage recording

Patch clamp electrodes were fabricated from borosillicate glass pulled in 7 steps using a horizontal puller (P-97, Sutter Instruments). Electrodes were filled with internal recording solution containing (in mM): K-gluconate 117, KCl 13, MgCl_2_ 1, CaCl_2_ 0.07, EGTA 0.1, HEPES 10. The final tip resistance of patch electrodes was 6–11 MΩ. Recordings were conducted in neurons with resting membrane potential between − 50 and − 75 mV and series resistance between 10 and 25 MΩ. Macropatch recordings were fabricated from borosilicate glass pulled in 7 steps and were filled with filtered aCSF. The final tip resistance was 200 kΩ. Whole-cell current clamp recordings were made using a Multiclamp 700B amplifier (Molecular Devices). Macropatch recordings were amplified and filtered (100–1000 Hz; Grass model P511s).

### Data acquisition

The preparation was placed in a recording chamber optimized for optical recording (JG 23 W/HP; Warner Instruments, Hamden CT), mounted on an upright microscope (Axioskop 2 FS; Zeiss Instruments, Jena, DE). The preparation was perfused at 4 ml/min with aCSF warmed to 24°–27° C and aerated with a 95%–5% O_2_–CO_2_ gas mixture. Respiratory motor output was recorded via suction electrode placed on ventral roots C2-C4. Optical signals, recorded at 10X, 20X, or 40X (Achroplan 10x/0.3 W Ph1, Achroplan 20x/0.5W, Achroplan 40x/0.75W; Zeiss), were recorded using a large format (18.7 mm diagonal, 11 μm × 11 μm pixels) sCMOS camera (Prime 95B, Teledyne Photometrics, Tucson AZ) that sampled a 1300 μm × 1300 μm (10X) field of view, with pixels capturing photons over 2.2 μm, 1.1 μm, and 0.55 μm of tissue (for 10X, 20X and 40X respectively; 2X2 binning). Synchronized image acquisition across the field of view was achieved by triggering the LED lamp (LCS-0470–50-48; Mightex, Ontario, Canada) with a square pulse that went high at the acquisition onset of the last sCMOS sensor line to be read and went low before the termination of the first sensor line acquisition. Light intensity was controlled by square pulse amplitude. High speed (100 Hz) optical recordings were made from a subarray spanning less than half of the sensor, with 3 ms light pulses, at maximal brightness; baseline recordings were carried out at 20 Hz, with 8–18 ms light pulses, at the lowest light intensity that enabled adequate signal quality; following synaptic blockade, faint optical signals were obtained at slower (10 Hz) and longer (50 ms) light pulses. The square wave that controlled the lamp, as well as population recording of respiratory drive was digitized at 20 kHz (PCI-6221, National Instruments, Austin TX). Camera control, voltage, and image acquisition was integrated in custom software (LabView; National Instruments, Austin TX) that synchronized all processes using the clock controlling voltage acquisition. In experiments in which patch or macropatch recordings were carried out, signals were digitized at 20 kHz (Powerlab/35; AD Instruments, Oxford UK) and stored using LabChart (AD Instruments). Voltage recordings stored in LabChart were synchronized with optical recordings using the digitized square wave that controlled the LED lamp.

### Stimulation

To evaluate synaptic blockade patency, a bipolar electrode (Pt:Ir, MicroProbes) was positioned roughly 250 μm ventral from the dorsal edge of the exposed face of the SSRH preparation, roughly 300 μm caudal to VIIn. Stimuli were applied (20 Hz train, 5 V, 2 ms pulses; S88 84T50D, Grass Instruments) to elicit post-synaptic activation of orofacial-regulatory interneuron populations dorsomedial to VIIn, recorded optically. The electrode was left in place, and following application of blocker cocktail, stimuli were repeated.

### Experimental protocol

At the start of each experiment, 100–150 s of baseline activity was recorded at × 10 magnification and 20 Hz; in some experiments, subsequent recordings under baseline conditions were made at the level of PiCo, pFRG/RTN, and preBötC at 20X or 40X. In the first set of experiments in which long, high-sampling-rate recordings were made, following selection of a subarray large enough to capture the VRC in the field of view, but small enough to enable acquisition at 100 Hz, a single long, high-speed recording was made. In some experiments in which blocker cocktail or MFA was applied, recordings were carried out over 10 min at 10 Hz, starting just before drug wash-in to record the transition to steady-state. In other experiments, after drugs were perfused into the recording chamber, the preparation was allowed to stabilize for at least 5 min, then activity was recorded for 2–10 min at 10 Hz and 10X, 20X, or 40X, followed by 20X recordings from the same networks recorded under baseline conditions. In experiments in which blocker cocktail, then MFA were applied, steady-state recordings under blocker cocktail were first carried out, then longer recordings were made of changes in activity before, during, and after MFA wash-in.

Unless otherwise noted, all computation described in the sections that follow were implemented in Matlab.

### Data analysis

Regions of interest capturing neuronal and glial Ca^2+^ transients were generated using semi-automated methods [15] implemented in LabView (National Instruments, Austin TX). Briefly, each image was subtracted from the image obtained 1.7 s earlier to generate a differenced image that set static or slowly varying pixels to zero, highlighting fast luminance fluctuations. These differenced images were binarized to set values far from the mean to white and values close to the mean to black. Binarized images were averaged, and regions of interest were extracted using waterfall thresholding. Signals were extracted by applying ROIs to the raw image series and filtered to remove slow luminance changes using a high-pass filter (zero phase-shift bidirectional Butterworth high-pass order 4, cutoff frequency 0.05 Hz).

Methods for processing optical and voltage recordings are described in detail elsewhere [8]. Briefly, to estimate signal-to-noise, signals were low-pass filtered (zero phase-shift bidirectional Butterworth high-pass order 4, cutoff frequency 5 Hz), and the residual of the low-pass-filtered and raw trace was expressed in dB; traces with dB < -6 were excluded from further analysis. As can be seen in the accompanying videos, activity along the VRC showed strong respiratory modulation; thus, almost all regions of interest were phase-locked to motor output. Respiratory drive recorded from C2-C4 was down-sampled to optical recording rates by taking the average of the absolute values of ventral root samples acquired during the high phase of the square pulse that controlled the lamp. Onset times of both motor output and respiratory network activity were estimated by first identifying a point reliably in a burst (mean + 6 X STD), then working backwards from this point to find the inflection point (defined as mean + 2 X STD). Each onset of the motor output defined a breath. For each breath, the lag for neural activity onset was estimated in relation to motor output onset time. For each neuron, a vector of lags was therefore obtained, one lag for each breath. The link between a pair of lag vectors was measured thanks to the non-parametric Kendall’s correlation coefficient Tau [58]. Also, for a given breath, lags of all neurons recorded could therefore be ordered and converted to ranks. To check for variability among breaths, we applied a principal component analysis [59] to the matrix of rank vectors for each breath.

To probe for the existence of functional anatomical parcellation along the VRC, a map of the anatomical distribution of the 10% neurons with earliest lags was generated. A lag for a neuron was defined as the median lag among all breaths. The early lag neurons were counted over a window (160 μm × 160 μm) and assigned to a 10 μm × 10 μm square centered on the window; the window was displaced in 10 μm steps vertically and horizontally. No fewer than 15 neurons were found in any window.

To analyze spectral features of syncytial rhythms, we computed the spectrogram of signals using sliding time windows of 30 s, displacements of 0.5 s, and 1024 frequencies in the range 0.05–3 Hz. The resulting spectrogram was smoothed using convolution by uniform windows of 11 × 3 points. Classical coherence between ROS and CAU signals was computed with a frequency resolution of 0.02 Hz using time windows of 30 s and displacements of 1 s. Directed coherence ROS and CAU signals were estimated using ARX time series models of order 100 for each signal. To obtain a significance level, we computed DCOH between the same ROS and CAU signals where one was time-reversed (which preserves the spectrum but breaks the temporal relationship). For each DCOH spectrum computed, we took the 95% percentile of DCOH values across the range [0.08–1] Hz as a limit for the confidence interval for the regular DCOH peak value between ROS and CAU signals.

To obtain maps of cross-correlation using pixel data, we first smoothed each image with a uniform 21 × 21 pixel window then bandpass filtered the signals between 0.4 Hz and 1.5 Hz. We then computed cross-correlations between a reference pixel and all other pixels, using the center of mass of rostral and caudal ROIs as reference.

To quantify burst onset times from macropatch recordings, pseudo-spikes were detected by thresholding the macropatch signal (criterion + -3 SD of the whole signal with a refractory period of 1.2 ms). Pseudo-spike times were then convolved with a Gaussian window (SD = 3.7 ms) to obtain a pseudo-firing rate time signal reflecting the local summed potential of the area. Relative burst onset times were estimated using 2 methods, first by cross-correlating the pseudo-spike firing rates obtained from paired macropatch recordings and second by aligning burst onsets (thresholded at 33% of the maximum pseudofiring rate).

### Immunohistochemistry

At the end of experiments, brainstems were stored overnight in 4% paraformaldehyde, then transferred to 1X phosphate-buffered saline (PBS). To preserve anatomical landmarks enabling the alignment of fixed and fresh tissue, a ~ 400 μm section was cut from the sagittal face of each preparation. Sections were washed in PBS with 0.1% triton X-100, blocked in 10% heat-inactivated normal donkey serum, incubated for 2 h in blocking buffer, incubated overnight in primary antibodies targeting somatostatin (1:500, Invitrogen PA5-85,759), choline acetyltransferase (1:200, Millipore AB144P), and phox2b (1:100, Santa Cruz sc-376997).

The following day, specimens were washed, transferred to secondary antibody for 2 h, and then imaged using a Nikon C2 Confocal microscope (Nikon Instruments, Melville, NY). Levels were modified and filtered for clarity using ImageJ (National Institutes of Health, Bethesda, MD).

## Supplementary Information

**Additional file 1: Video**: Optical recording of baseline network activity accompanying spontaneous fictive respiration; Sampling rate 20 Hz. Initially, unprocessed video is shown to facilitate identification of anatomical landmarks, thereafter images are subtracted from the image obtained 1.7 s earlier to increase the salience of Ca2+ transients. In the second half of the video, the sagittal face of the same preparation is shown following immunohistochemical processing to label phox2b (expressed in pFRG and other neurons), somatostatin (expressed along the VRG and preBotC), and choline acetyltransferase (expressed on premotoneurons). Available at: https://figshare.com/articles/media/Gourevitch_et_al_2023/21901119

**Additional file 2: Video**: High-speed optical recordings provide evidence for synchronous activation along the VRC at inspiratory onset (P4 mouse). The video consists of the following elements: 1. Five inspiratory bursts acquired at 10 X, 100 Hz, slowed to 10 fps, with gaps during expiration; ROIs are superimposed on the last burst. Available at: https://figshare.com/articles/media/SV2_190902_2_100_Hz_20_fps_playback_avi/21901143

**Additional file 3: **Animation: Animation of onset times (earliest in black; latest in yellow) of anatomically dispersed ROIs along the ventral respiratory column extracted from the dataset shown in S2. 2. Available at: https://figshare.com/articles/media/SV_3_onset_sequence_190902_2_avi/21901179

**Additional file 4: Figure**: Top: Rectified macropatch traces acquired at 20 kHz, and downsampled to 4 kHz. Bottom: pseudospikes extracted from rectified data via thresholding as described in methods. Left panels contain recordings from the caudal macropatch electrode, right panels contain recordings from the rostral macropatch electrode.

**Additional file 5: Video**: Loss of post-synaptic swallow network activation following synaptic blockade confirms disruption of synaptic transmission by blocker cocktail in networks close to the slice surface (P4 mouse, 0:26). The video consists of the following elements: 1. Raw image sequence of one inspiratory burst and fictive swallow in the intact network. 2. Differenced image sequence of inspiratory burst and fictive swallow in the intact network. Note strong post-synaptic activation of networks dorsal to VIIn. 3.Differenced image sequence of stimulus-induced network activation following synaptic blockade. Here post-synaptic activity is largely absent, confirming blockade patency. Available at: https://figshare.com/articles/media/SV4_210617_block_validation_mp4/21901188

**Additional file 6: Video**: Synchronized syncytial rhythmic activity overlaps with respiratory network activity The video consists of the following elements: 1. View of the preparation with VIIn outlined. 2. Differenced view of baseline activity over 3 respiratory cycles (10X, 20Hz, 18 ms exposure); regions of interest are superimposed over the final inspiratory burst. 3. Averaged differenced inspiratory activity with ROIs superimposed. 4. Differenced view of syncytial activity over 5 cycles (10X, 10Hz, 50 ms exposure); two large ROIs used to generate traces in **Supplemental Fig. 2** are superimposed over the last syncytial activation wave. Available at: https://figshare.com/articles/media/SV4_2102152_2_syncytial_video_mp4/21901209

**Additional file 7: Figure**: ROIs and associated traces from dataset shown in **Supplemental Video 4**. Top: View of differenced inspiratory activity, averaged over 5 breaths. ROIs extracted from the intact network are shown in yellow, field-potential ROIs obtained following synaptic blockade are shown in red and green. Middle: traces associated with control ROIs over a 100 s interval. Rectified integrated motor output is shown at the top. Bottom: traces associated with caudal (green) and rostral (red) field potential ROIs, recorded over 100 s following synaptic blockade. These traces show features common to many experiments: the rhythmic activity is phase-locked and faster than respiratory rhythm in the intact network; peaks from the caudal ROI’s traces are bimodally distributed, while peaks from the rostral ROI are unimodal, consistent with non-linear amplification of syncytial drive by constituents of caudal networks

**Additional file 8: Figure**: A. Optical recording of network activity from an inducible-cre dbx1-GCaMP6F mouse exposed to tamoxifen exposed to tamoxifen at E9.5 to bias expression toward neurons. Prior to blocker cocktail wash-in, Ca^2+^ transients phase-locked to inspiratory drive are recorded, and following synaptic blockade, Ca^2+^ transients display syncytial oscillations. B. In an inducible cre dbx1-GCaMP6F mouse injected with tamoxifen at E12.5 in order to bias GCaMP6F expression toward glia, strong respiration-modulated activity is present under baseline conditions (left), but fallowing synaptic blockade, syncytial oscillation are absent. These findings support the conjecture that syncytial oscillations are not expressed in glia specified by *dbx1*

## Data Availability

All data generated or analyzed during this study are included in this published article, its supplementary information files, and publicly available repositories. The optical recordings carried out in this study generated more than 4 TB of data. In consultation with the editors, it was agreed that representative data rather than all the image series would be uploaded to a repository. To this end, the image series used to generate the videos Additional file 2 and Additional file 6 have been uploaded as zipped folders to FigShare: https://figshare.com/articles/dataset/20190902-3_100_Hz_10X_ventrolateral_medulla_respiratory_activity_image_series/22290430https://figshare.com/articles/dataset/2_15_2021-4_06_PM-16_7z/22290451.
