# Author Correction: Synchronization of inspiratory burst onset along the ventral respiratory column in the neonate mouse is mediated by electrotonic coupling

**DOI:** 10.1186/s12915-023-01615-0

**Published:** 2023-05-15

**Authors:** Boris Gourévitch, Teresa Pitts, Kimberly Iceman, Mitchell Reed, Jun Cai, Tianci Chu, Wenxin Zeng, Consuelo Morgado-Valle, Nicholas Mellen

**Affiliations:** 1 Institut Pasteur, Université Paris Cité, Inserm, Institut de L’Audition, 75012 Paris, France; 2 CNRS, Paris, France; 3 Department of Neurological Surgery, University of Louisville, Louisville, KY USA; 4 Department of Pediatrics, University of Louisville, Louisville, KY USA; 5 Instituto de Investigaciones Cerebrales, Universidad Veracruzana, Xalapa, Veracruz, México; 6 Department of Neurology, University of Louisville, Louisville, KY USA

**Correction: BMC Biol 21, 83 (2023)**

**https://doi.org/10.1186/s12915-023-01575-5**

Following publication of the original article [1], the authors identified errors in the affiliations of Boris Gourévitch which are corrected as follows:

1) Institut Pasteur, Université Paris Cité, Inserm, Institut de l’Audition, F-75012 Paris, France

2) CNRS, Paris, France

The authors also omitted two funding sources which they would like to state ahead:

Fondation pour l’Audition FPA-IDA03 (BG).

French National Research Agency ANR-21-CE34-0012 (BG).
