# Supplementary figures and images for: Synchronization of inspiratory burst onset along the ventral respiratory column in the neonate mouse is mediated by electrotonic coupling
Source: BMC Biol. 2023 Apr 15;21:83. doi: 10.1186/s12915-023-01575-5 (PMC10105963; doi:10.1186/s12915-023-01575-5)

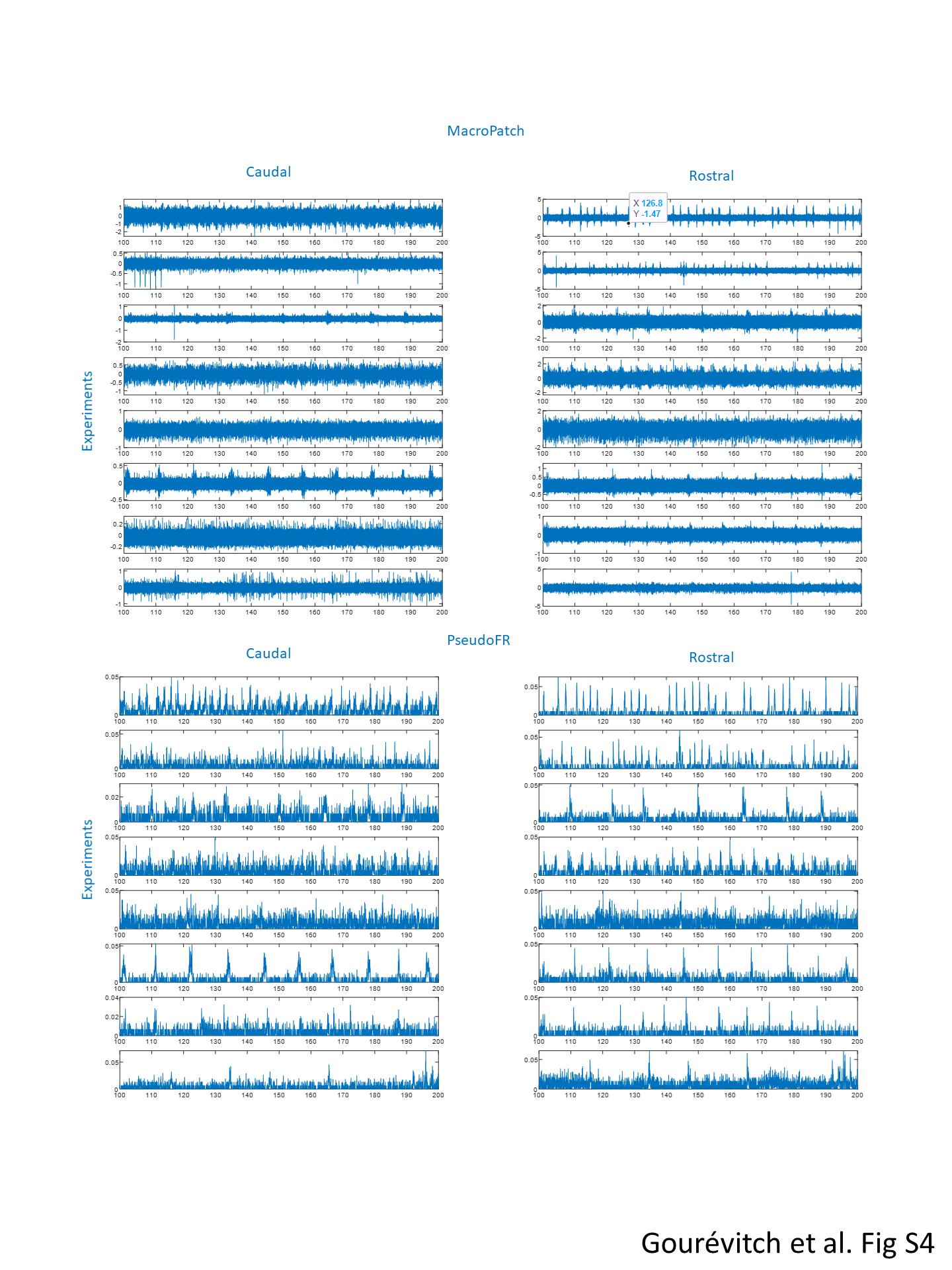

Supplement: Supplementary file 4 — Additional file 4: Figure: Top: Rectified macropatch traces acquired at 20 kHz, and downsampled to 4 kHz. Bottom: pseudospikes extracted from rectified data via thresholding as described in methods. Left panels contain recordings from the caudal macropatch electrode, right panels contain recordings from the rostral macropatch electrode. [file 12915_2023_1575_MOESM4_ESM.png]

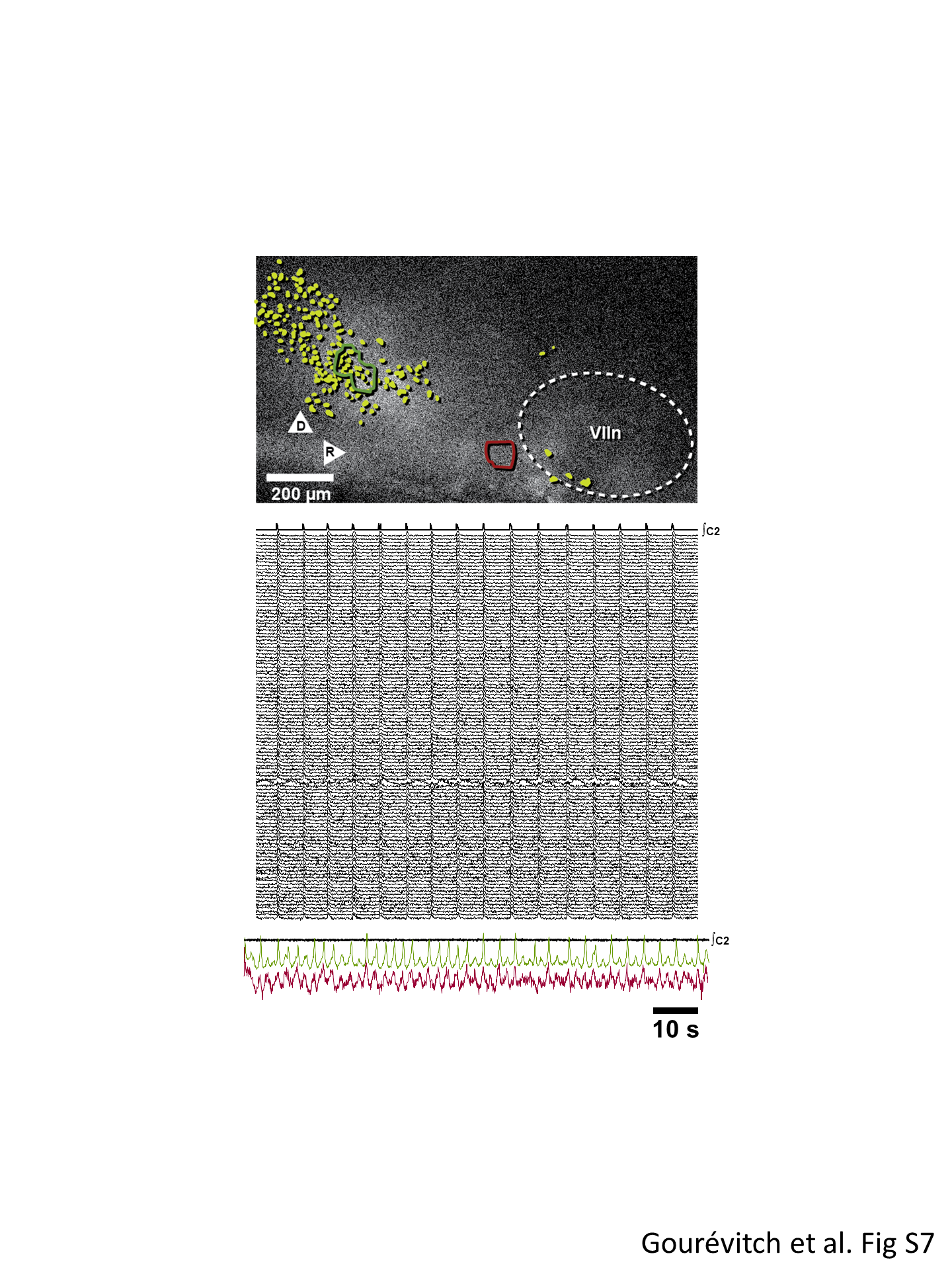

Supplement: Supplementary file 7 — Additional file 7: Figure: ROIs and associated traces from dataset shown in Supplemental Video 4. Top: View of differenced inspiratory activity, averaged over 5 breaths. ROIs extracted from the intact network are shown in yellow, field-potential ROIs obtained following synaptic blockade are shown in red and green. Middle: traces associated with control ROIs over a 100 s interval. Rectified integrated motor output is shown at the top. Bottom: traces associated with caudal (green) and rostral (red) field potential ROIs, recorded over 100 s following synaptic blockade. These traces show features common to many experiments: the rhythmic activity is phase-locked and faster than respiratory rhythm in the intact network; peaks from the caudal ROI’s traces are bimodally distributed, while peaks from the rostral ROI are unimodal, consistent with non-linear amplification of syncytial drive by constituents of caudal networks [file 12915_2023_1575_MOESM7_ESM.png]

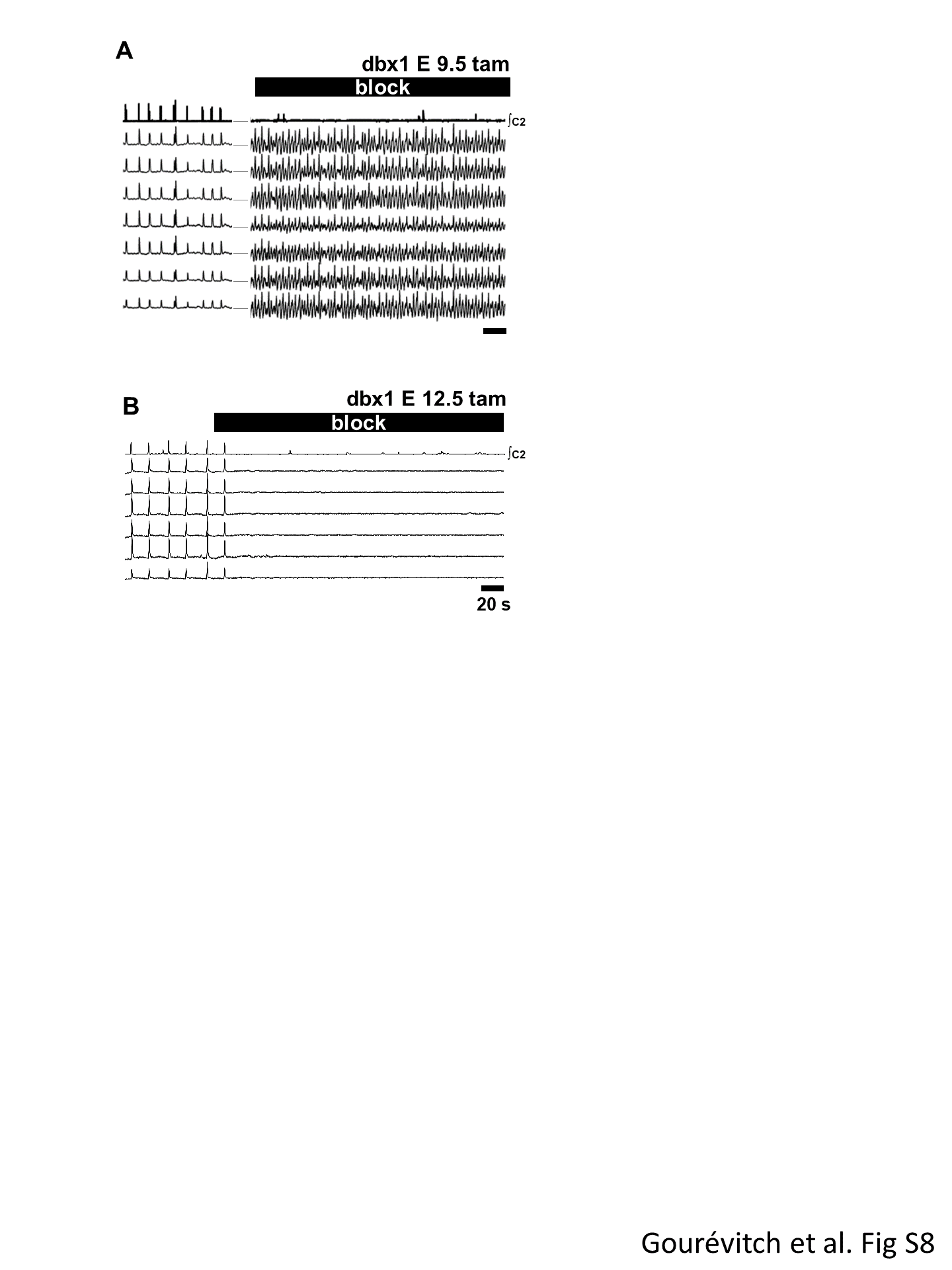

Supplement: Supplementary file 8 — Additional file 8: Figure: A. Optical recording of network activity from an inducible-cre dbx1-GCaMP6F mouse exposed to tamoxifen exposed to tamoxifen at E9.5 to bias expression toward neurons. Prior to blocker cocktail wash-in, Ca2+ transients phase-locked to inspiratory drive are recorded, and following synaptic blockade, Ca2+ transients display syncytial oscillations. B. In an inducible cre dbx1-GCaMP6F mouse injected with tamoxifen at E12.5 in order to bias GCaMP6F expression toward glia, strong respiration-modulated activity is present under baseline conditions (left), but fallowing synaptic blockade, syncytial oscillation are absent. These findings support the conjecture that syncytial oscillations are not expressed in glia specified by dbx1 [file 12915_2023_1575_MOESM8_ESM.png]
